# Therapy-Induced Changes in CXCR4 Expression in Tumor Xenografts Can Be Monitored Noninvasively with N-[^11^C]Methyl-AMD3465 PET

**DOI:** 10.1007/s11307-019-01447-x

**Published:** 2019-12-04

**Authors:** SV Hartimath, O. Draghiciu, T Daemen, H.W. Nijman, A. van Waarde, R.A.J.O. Dierckx, E.F.J. de Vries

**Affiliations:** 1 Department of Nuclear Medicine and Molecular Imaging, University of Groningen, University Medical Center Groningen, Hanzeplein 1, P.O. Box 31.001, 9713 GZ Groningen, The Netherlands; 2 Department of Medical Microbiology, Tumor Virology and Cancer Immunotherapy, University of Groningen, University Medical Center Groningen, Groningen, The Netherlands; 3 Department of Gynecology, University of Groningen, University Medical Center Groningen, Groningen, The Netherlands

**Keywords:** CXCR4 expression, Treatment monitoring, Cervical cancer, Immunization, Radiotherapy, PET, Immune cell infiltration

## Abstract

**Purpose:**

Chemokine CXCL12 and its receptor CXCR4 are constitutively overexpressed in human cancers. The CXCL12-CXCR4 signaling axis plays an important role in tumor progression and metastasis, but also in treatment-induced recruitment of CXCR4-expressing cytotoxic immune cells. Here, we aimed to demonstrate the feasibility of N-[^11^C]methyl-AMD3465 positron emission tomography (PET) to monitor changes in CXCR4 density in tumors after single-fraction local radiotherapy or in combination with immunization.

**Procedure:**

TC-1 cells expressing human papillomavirus antigens E6 and E7 were inoculated into the C57BL/6 mice subcutaneously. Two weeks after tumor cell inoculation, mice were irradiated with a single-fraction 14-Gy dose of X-ray. One group of irradiated mice was immunized with an alpha-viral vector vaccine, SFVeE6,7, and another group received daily injections of the CXCR4 antagonist AMD3100 (3 mg/kg -intraperitoneal (i.p.)). Seven days after irradiation, all animals underwent N-[^11^C]methyl-AMD3465 PET.

**Results:**

PET imaging showed N-[^11^C]methyl-AMD3465 uptake in the tumor of single-fraction irradiated mice was nearly 2.5-fold higher than in sham-irradiated tumors (1.07 ± 0.31 %ID/g vs. 0.42 ± 0.05 % ID/g, *p* < 0.01). The tumor uptake was further increased by 4-fold (1.73 ± 0.17 % ID/g vs 0.42 ± 0.05 % ID/g, *p* < 0.01) in mice treated with single-fraction radiotherapy in combination with SFVeE6,7 immunization. Administration of AMD3100 caused a 4.5-fold reduction in the tracer uptake in the tumor of irradiated animals (0.24 ± 0.1 % ID/g, *p* < 0.001), suggesting that tracer uptake is indeed due to CXCR4-mediated chemotaxis.

**Conclusion:**

This study demonstrates the feasibility of N-[^11^C]methyl-AMD3465 PET imaging to monitor treatment-induced changes in the density of CXCR4 receptors in tumors and justifies further evaluation of CXCR4 as a potential imaging biomarker for evaluation of anti-tumor therapies.

## Introduction

CXCR4 is a seven-transmembrane G-protein coupled receptor, which is overexpressed by stromal cells and tumor cells in more than 20 different human cancers types [1]. CXCR4 is involved in various biological processes, including immune cell trafficking, tumor growth, and metastasis [2–7]. CXCR4 signaling is mediated by stromal derived factor-1α (CXCL12) and leads to G-protein-mediated activation of downstream signaling pathways via transcription factors that promote cell proliferation, cell survival, angiogenesis, invasion, and cell migration [8, 9].

Migration of cancer cells is directly dependent on the interactions between cell surface molecules on the tumor cells, like CXCR4, and the release of chemokines, like CXCL12, by tissues that are targets for metastases. It is believed that organs with high levels of CXCL12, such as lymph nodes, lungs, liver, and bones, are the first destination of metastatic tumor cells expressing CXCR4 receptors. This hypothesis was supported by animal studies, showing that CXCR4-positive tumor cells migrated from their primary region to these CXCL12 secreting organs [10, 11]. The CXCR4-CXCL12 signaling pathway, however, is not only involved in the migration of cancer cells, but also in the trafficking of stem cells and immune cells, such as CXCR4-expressing hematopoietic stem cells, progenitor cells, pre-B lymphocytes, and T lymphocytes [12, 13]. Increased secretion of CXCL12 by the tumor, for example, as a result of hypoxia or treatment, stimulates the infiltration of CXCR4 expressing immune cells [14, 15].

High expression of CXCR4 by tumor cells has been associated with treatment resistance [16, 17]. Recent data indicate that standard chemotherapeutic agents and radiotherapy can induce dynamic changes in the surface expression of CXCR4. This therapy-induced overexpression of CXCR4 was suggested to be involved in acquired therapeutic resistance [16, 17]. Moreover, mutations in the CXCR4 gene that lead to overexpression of CXCR4 receptors were found to induce resistance towards conventional therapy [17, 18]. Inhibition of the CXCR4-CXCL12 axis, on the other hand, can sensitize cancer cells to chemotherapy or radiotherapy by inhibiting the interaction between the CXCR4-expressing tumor cells and stromal cells, resulting in decreased cancer cell protection by the CXCL12 releasing stromal cells [19–21]. When radiotherapy or chemotherapy of solid tumors was combined with the administration of a CXCR4 antagonist such as AMD3100, a significant decrease in primary tumor volume and reduced metastatic burden was observed [21–24]. The use of anti-CXCR4 drugs can also potentiate the anti-tumor activity of several targeted drugs, such as tyrosine kinase inhibitor or anti-PD-1 and anti-CTLA4 antibodies [25–28].

Since the CXCR4-CXCL12 signaling pathway plays an important role in oncogenesis, treatment-induced resistance and immune cell trafficking, CXCR4 could be an interesting biomarker to predict outcome and monitor treatment response. We recently developed N-[^11^C]methyl-AMD3465 as a new positron emission tomography (PET) tracer for imaging of CXCR4 receptors [29, 30]. In vivo evaluation of this tracer showed favorable receptor binding, biodistribution, and pharmacokinetics for imaging [29, 30]. The aim of the current study is to demonstrate the feasibility of N-[^11^C]methyl-AMD3465 PET to monitor treatment-induced changes in the density of CXCR4 receptors in the tumor. In particular, we investigated the effect of a single-fraction radiotherapy, as an example of a conventional treatment, and cancer immunization [32–34], as an example of an experimental immunotherapy. In addition, tumor-bearing mice were treated with the CXCR4 antagonist AMD3100 (Plerixafor®) to inhibit the chemotaxis mediated by the CXCR4 receptor.

## Materials and Methods

### General

**Fig. 1.**
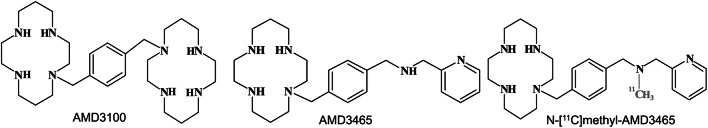
Structure of AMD3100, AMD3465, and N-[^11^C]methyl-AMD3465.

All chemicals and reagents were obtained from commercial suppliers and used without further purification. The drug AMD3100 octahydrochloride (AMD3100.8HCl; Plerixafor®) was prepared as described in the literature (Fig. 1) [29]. A stock solution of Plerixafor® was prepared in phosphate-buffered saline (PBS), and the pH of the solution was adjusted to neutral with 1 M NaOH (Fig. 1). The radiotracer N-[^11^C]methyl-AMD3465 was prepared as previously described (Fig. 1) [30]. The TC-1 cell line was created from C57BL/6 primary lung epithelial cells by transfection with a retroviral vector that expresses a fusion protein of the HPV16 early genes E6 and E7 [32]. Cells were cultured as previously described [33]. The production and quality control of the Semliki Forest virus vector SFVeE6, 7 for immunization was performed as previously described [33].

### Animal Model

All animal experiments were performed in the compliance with the Dutch law on Animal experiments. The Institutional Animal Care and Use Committee of the University of Groningen (DEC6073E) approved the protocol. Specified pathogen-free female C57BL/6 mice between the age of 8 and 14 weeks were used (Harlan CPB, The Netherlands). Mice were maintained at a 12 h/12 h day/night regimen and fed standard laboratory chow. Mice were subcutaneously inoculated in the neck region with 2 × 10^4^ TC-1 cells suspended in 0.05 ml Hank’s balanced salt solution (Invitrogen, Paisley, UK). The animals were randomly divided into four groups, which received the following treatments: (1) sham-irradiation (control, *n* = 5), (2) a local single-fraction of 14-Gy tumor irradiation (*n* = 6), (3) a single-fraction 14-Gy tumor irradiation followed by immunization with SFVeE6,7 (*n* = 6), and (4) the last group received a single-fraction 14-Gy tumor irradiation followed by treatment with the CXCR4 antagonist AMD3100.8HCl (3 mg/kg i.p., *n* = 5). At the end of the study, animals were euthanized, the tumor was harvested, and tumor weight was measured before snap-freezing.

### Treatments

Two weeks after tumor cell inoculation, mice were anesthetized with isoflurane and placed in plastic constrainers to ensure immobilization for the localized irradiation of the tumor. TC-1 tumors were subjected to a local single-fraction 14-Gy dose of X-ray irradiation, using an X-RAD 320 Biological Irradiator (Precision X-Ray, North Branford, CT, USA). The X-ray delivery rate was 1.64 Gy/min (1 Gy/min at 320 kV, 12.5 mA, 50 cm SSD (HVL ≈ 4 mm Cu)). Sham-irradiated animals underwent the same procedure, but the irradiation equipment remained switched off. One day after irradiation, one group (irradiated only) received a vehicle injection (PBS), the second group of mice received a single dose intramuscular injection of 5 × 10^6^ SFVeE6,7 particles (irradiation + immunization group), and the last group received daily intraperitoneal (i.p.) injections of AMD3100.8HCl (3 mg/kg) until the end of the experiment (6 days). The purpose of AMD3100 treatment in this study is to block the CXCR4 dependent chemotaxis by saturation of the CXCR4 receptors.

### PET Acquisition

PET imaging experiments were performed 7 days after irradiation. Mice were anaesthetized with isoflurane (5% induction; 2% for maintenance) in medical air. Two animals were placed in the prone position on a home-made Perspex “bunk bed” in the PET camera (microPET Focus 220; Siemens Medical Solution USA) with the tumors in the field of view. Animals were injected with 20 ± 2 MBq of N-[^11^C]methyl-AMD3465 (0.45 ± 0.15 nmol) via the tail vein, and the acquisition of a 30-min dynamic PET scan was started immediately. After the emission scan was complete, a transmission scan of 900 s with a Co-57 point source was obtained for the correction of attenuation and scatter by tissue.

### Image Reconstruction

All the emission scans were normalized and corrected for attenuation, scatter, and radioactive decay. Emission sinograms were iteratively reconstructed using an ordered subset expectation maximization (OSEM) algorithm with 4 iterations and 16 subsets. The final dataset consists of 6 frames of 5 min, each containing 24 transverse slices with a slice thickness of 0.8 mm and an in-plane 128 × 128 image matrix with a pixel size of 1.1 mm. In order to have a better signal-to-noise ratio and image quality, summed PET images were used to draw volumes of interest (VOIs). These VOIs were used for quantitative PET measurements of tracer uptake using only the last frame (i.e., frame-6, 25–30 min). To avoid partial volume effects, conservative VOIs were drawn and the maximum tracer concentration in the VOI (in Bq/ml) was quantified using Inveon standard software (Inveon, Siemens, USA). The maximum tracer concentration in tissue (C_t_(max) - Bq/ml) was normalized to the injected activity (D_inj_ – Bq) and multiplied by 100% to calculate the percentage of the injected dose per gram tissue (%ID/g): it was assumed that 1 ml of tissue corresponds to 1 g.

$$ \frac{\% ID}{g}=\frac{Ct\left(\mathit{\max}\right)}{Dinj}.100\% $$

### Immunohistochemistry

Immunohistochemistry (IHC) was performed as previously described [29–31]. Tumors were harvested, snap-frozen in liquid nitrogen, and stored in a freezer at − 80 °C until required for further use. For each tumor, 3–5 sections were prepared with a thickness of 5 μm. Then, the tissue was fixed in acetone followed by washing with 2 % hydrogen peroxide solution to block endogenous peroxidase activity. To reduce non-specific binding, the sections were incubated in 2.5% normal serum. Then, sections were incubated with primary rabbit polyclonal anti-CXCR4 antibody (Abcam, clone 2074; Cambridge, UK) at a dilution of 1:500 overnight at 4 °C. Subsequently, the sections were incubated with the secondary antibody, which was conjugated with horseradish peroxidase (HRP), and the tertiary antibody, as was described in the recommendations by the manufacturer (Dako, Belgium). The slides were stained diaminobenzidine (DAB), counterstained with hematoxylin and washed. Control experiments, in which the addition of primary antibody was eliminated, were performed to assess the extent of non-specific staining. The slides were examined under a microscope (Leica) at 10 different areas for each sample and scored according to the staining (0, no; 1, weak; 2, moderate; and 3, strong staining). Based on the intensity of staining, each area received a score and all areas were summed to give a total score for each slide. To avoid any error, an expert pathologist opinion was taken during reading the slides. The slides with improper tumor sections were excluded from the analysis; only slides with full tissues were included in the study. For statistical analysis, we included a minimum of three slides from each animal.

### Statistical Analysis

All data are expressed as mean ± standard deviation (SD). Statistical analyses by one-way ANOVA were performed using GraphPad Prism 5. Probability (*p*) values lower than 0.05 were considered statistically significant.

## Results

### CXCR4 Imaging in the Tumor

**Fig. 2.**
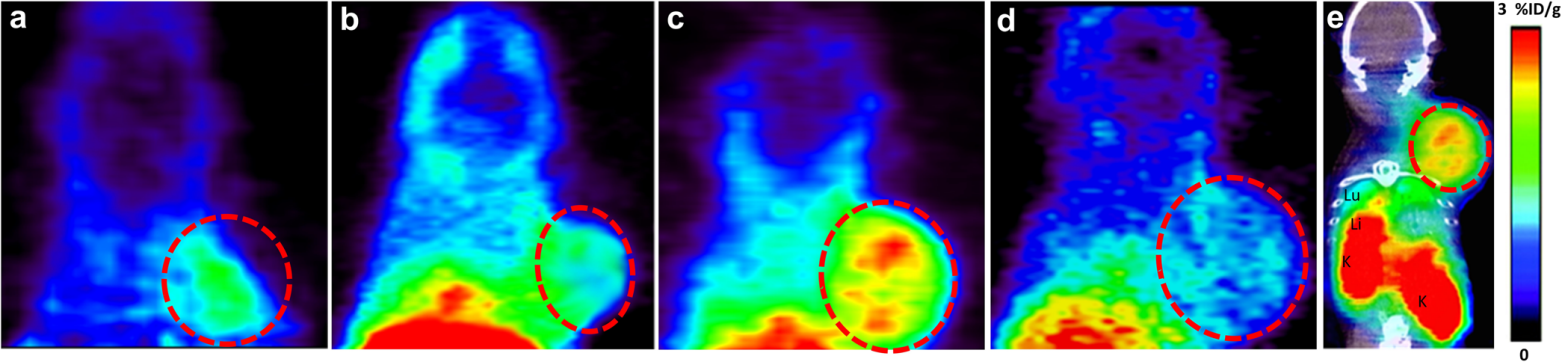
N-[^11^C]methyl-AMD3465 PET images (5–30 min) of female C57BL/6 mice bearing a TC-1 tumor in the neck. a Tumor-bearing mice were treated with a sham-irradiation, b single-fraction radiotherapy of 14 Gy on the tumor, c a single-fraction radiotherapy followed by immunization with a single dose of 5 × 10^6^ SFVeE6,7 particles, and d a single-dose irradiation followed by daily administration of AMD3100 (3 mg/kg, i.p). e PET-CT fusion image showing a tumor bearing mice treated with single-fraction radiotherapy along with immunization. The tumor was indicated by red dashed line. Lu lungs, Li liver, K kidney.

Three weeks after tumor inoculation, all animals underwent PET scanning. Figure 2 represents PET scans of the tracer uptake in the TC-1 tumor after different treatments. The tumors were clearly visualized and the uptake of tracer was found to be homogenously distributed within most of the tumors. Even in the sham-irradiated control group, the basal expression of CXCR4 in the tumor could be detected.

**Fig. 3.**
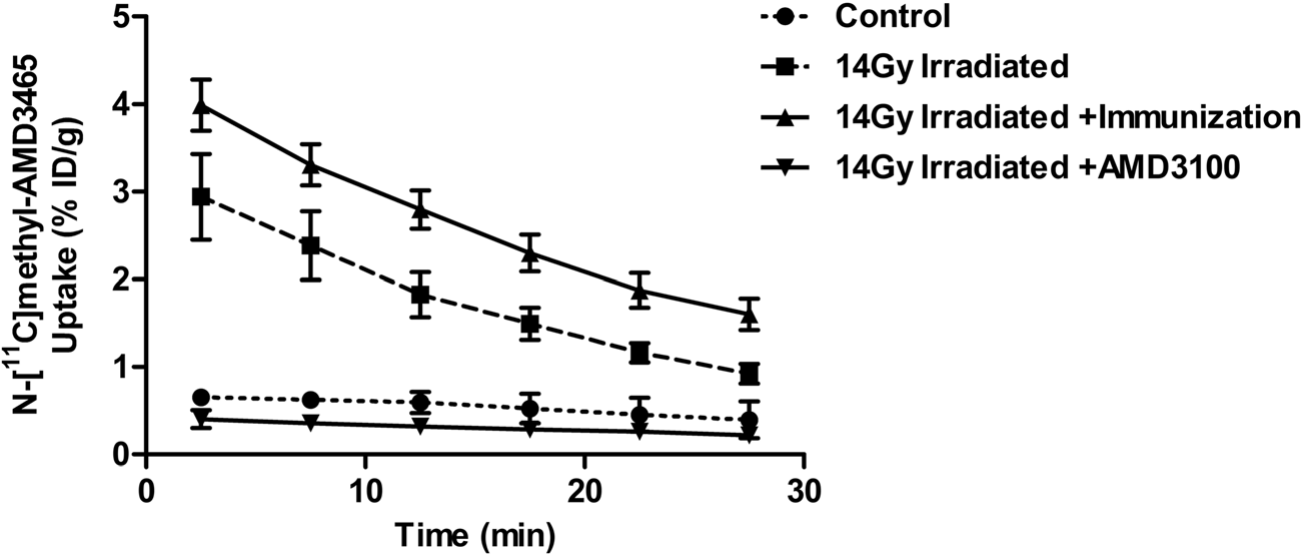
PET-derived time activity curves of N-[^11^C]methyl-AMD3465 (5–30 min) of the tumor of sham-irradiated (control mice, *n* = 5), a local single-fraction radiotherapy (14 Gy) irradiated mice (*n* = 6), single-fraction tumor irradiated in combination with SFVeE6,7 immunized mice (*n* = 6) or in mice upon local 14-Gy tumor irradiation followed by daily AMD3100 treatment (*n* = 5, 3 mg/kg, i.p). All TACs were converted to percentage ID per gram (%ID/g).

The time-activity curves (TACs) of the tumor indicated highest tumor uptake within 5 min after tracer injection followed by washout (Fig. 3). The area under the curve (AUC), calculated with the trapezoidal method, was approximate 3.5 times higher for the irradiated group when compared to the sham-irradiated control group (44 ± 2 % ID/(g min) vs 14 ± 1 % ID/(g min), *p* < 0.01). For animals that received irradiation in combination with immunization, the AUC was even 5-fold higher than for sham-irradiated controls (65 ± 1 % ID/(g min) vs. 14 ± 1 % ID/(g min), *p* < 0.01). In contrast, the AUC for the AMD3100-treated group was 5.6-fold lower when compared to the irradiated group (44 ± 2 % ID/(g min) vs. 8 ± 1 % ID/(g min), *p* < 0.01). Moreover, the AUC of the irradiated animals treated with AMD3100 was even 1.7-fold lower when compared to the sham-irradiated control group (*p* < 0.05).

**Fig. 4.**
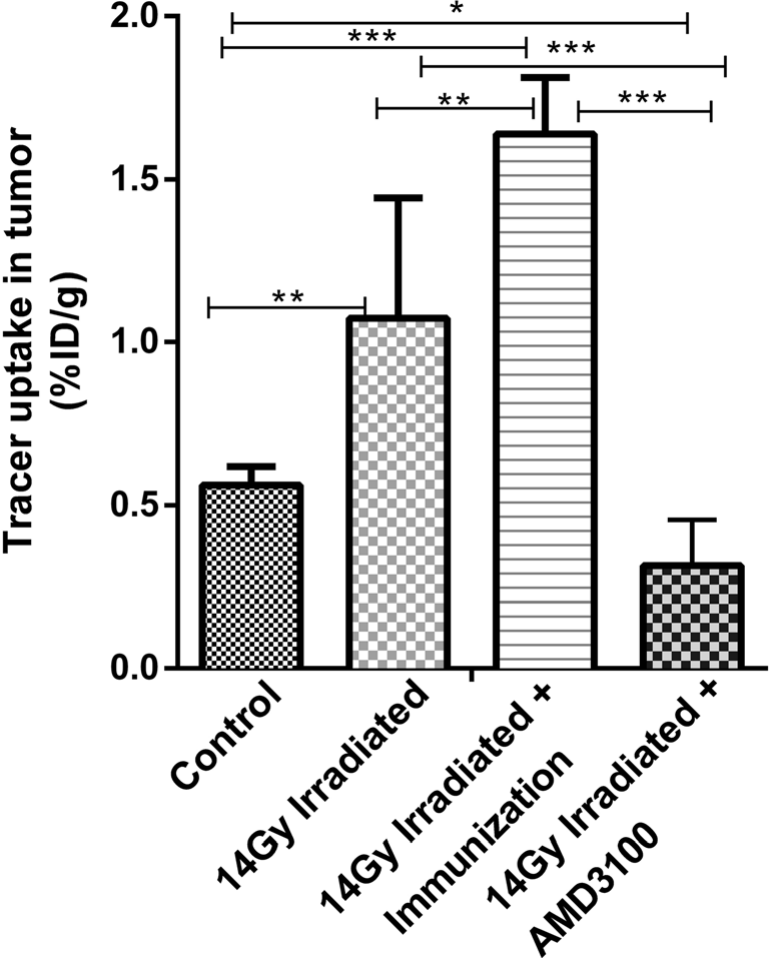
PET-derived tumor uptake of N-[^11^C]methyl-AMD3465 in mice treated with sham-irradiation (*n* = 5), with a local single-fraction radiotherapy (14 Gy, *n* = 6), or with local tumor irradiation in combination with immunization with a single dose of 5 × 10^6^ SFVeE6,7 (*n* = 6), or with local tumor irradiation in combination with daily AMD3100 treatment (*n* = 5, 3 mg/kg, i.p). Quantitive PET data was calculated from 25 to 30 min postinjection (frame 6). The tracer uptake was expressed as maximum percentage injected dose per gram tumor tissue (%ID/g). All bars represent the mean of the maximum %ID/g, and error bars represent standard deviations (SDs). Statistically significant differences are indicated by *p < 0.05, **p < 0.01, and ***p < 0.001).

The tumors were most clearly visualized 25–30 min after tracer injection. Figure 4 shows the quantitative N-[^11^C]methyl-AMD3465 uptake in the tumor, as determined from the 25–30 min frame 6 of the PET scan. Seven days after irradiation with a single-fraction radiotherapy of 14 Gy, the tracer uptake in the tumor was nearly 2.5-fold higher than in the sham-irradiated group (1.07 ± 0.31 % ID (*n* = 6)/g vs. 0.42 ± 0.05 % ID/g (*n* = 5), *p* < 0.01). When local tumor irradiation was combined with immunization with a single dose of SFVeE6,7 particles, tracer uptake in the tumor was even further increased by approximately 70%, when compared to mice that were treated with radiation alone (1.73 ± 0.17 (*n* = 6), *p* < 0.01). In contrast, administration of a daily dose of AMD3100 caused an almost 4.5-fold reduction in tracer uptake in the tumor of irradiated animals (0.24 ± 0.1 % ID/g (*n* = 5), *p* < 0.001). Moreover, tracer uptake in the tumor of animals treated with AMD3100 was significantly lower than uptake in tumors of sham-irradiated animals (57%, *p* < 0.05).

### Tracer Uptake in Major Organs

Apart from the tumor, organs such as liver, kidney, and heart were also clearly visible in the PET images (Fig. 2). Tracer uptake in the liver 25–30 min p.i. was nearly 15 times higher than in the tumor. Tracer uptake in the liver of mice receiving local tumor irradiation in combination with immunization was approximately 20% higher (12.5 ± 2.5 %ID/g), when compared to the other groups (~ 10 % ID/g), but this difference was not statistically significant. Furthermore, the kidneys (66 ± 12 % ID/g) and bladder (71 ± 24 % ID/g) were the organs with highest tracer uptake and therefore clearly visible in the PET images (Table 1). Kidneys and bladder uptake was not significantly different among the groups, indicating that renal clearance of the tracer was not significantly affected by the treatments. Although heart and spleen were visible in the PET images, quantification of tracer uptake in these organs was difficult, due to spillover from liver. Tracer uptake in the brain was low (0.5 % ID/g) and not significantly different among groups. Furthermore, the tumor from all animals were harvested at the end of the study to assess any treatment effects, but no significant differences in the tumor weight among the sham-irradiated, single-fraction radiotherapy, or in combination with immunization or AMD3100-treated groups were observed (0.86 ± 0.21 g vs 0.71 ± 0.13 g vs 0.65 ± 0.16g vs 0.74 ± 0.11 g, respectively, *p* > 0.05). This suggests that single-fraction radiotherapy alone or in combination with single-dose immunization or AMD3100 treatment did not induce any effect on the tumor size or tumor mass at the time of measurement.

**Table 1.** PET-derived N-[^11^C]methyl-AMD3465 tracer uptake in liver, kidney, bladder, and brain after different treatments. The tracer uptake is expressed as the maximum %ID/g. No statistically significant differences in tracer uptake between groups were observed

Organs	Control	14 Gy irradiated	14 Gy irradiated + immunization	14 Gy irradiation + AMD3100
Liver	9 ± 1	10 ± 2	12 ± 2	10 ± 2
Kidney	59 ± 12	62 ± 10	66 ± 12	60 ± 17
Bladder	69 ± 15	70 ± 18	71 ± 24	67 ± 26
Brain	0.5 ± 0.2	0.6 ± 0.1	0.7 ± 0.3	0.5 ± 0.2

### Immunohistochemistry (IHC)

**Fig. 5.**
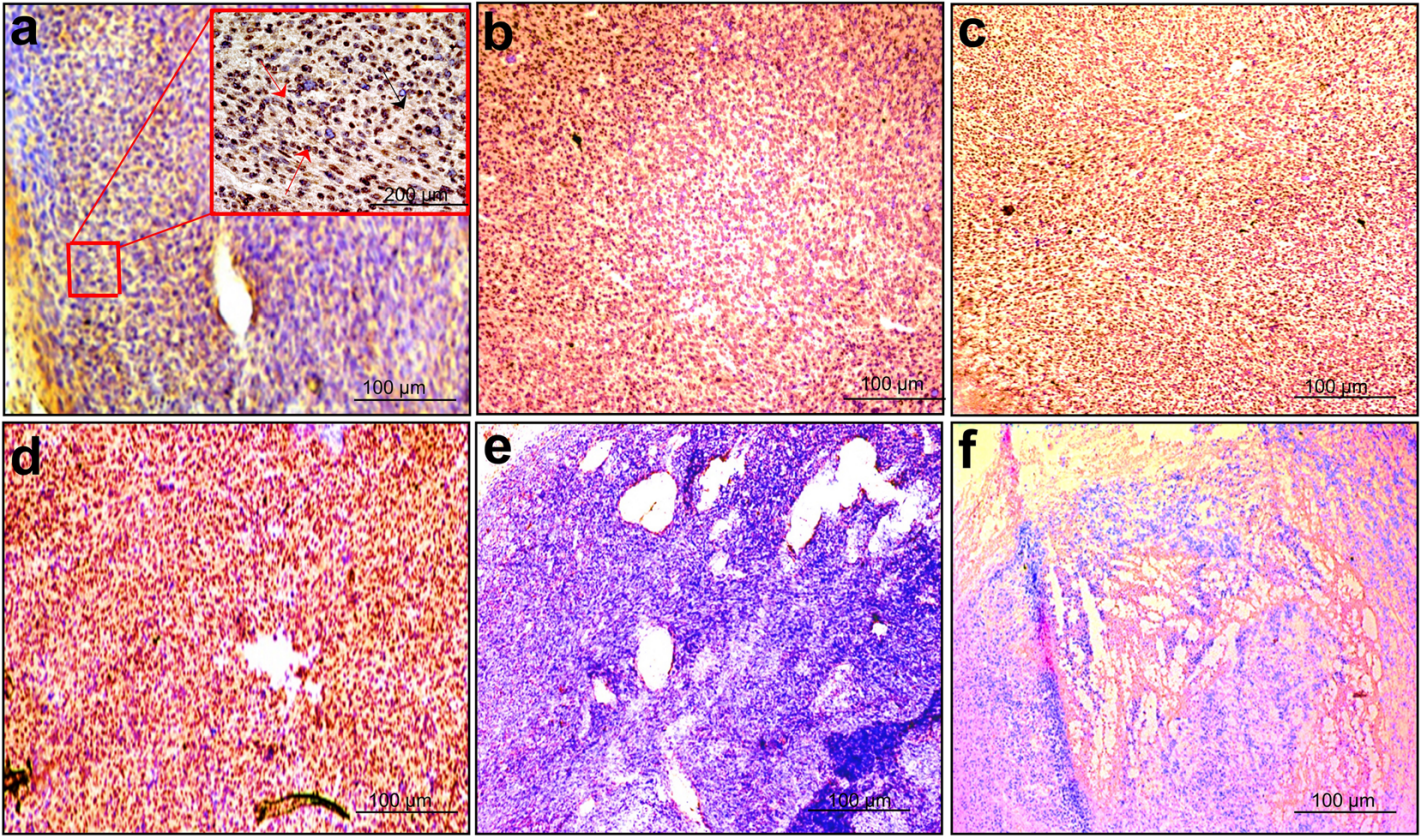
Immunohistochemistry of CXCR4 expression in TC-1 tumor sections. CXCR4 expression was monitored after different treatments: **a** sham-irradiated (*n* = 5). **b** A local single-fraction 14-Gy radiotherapy (*n* = 6). **c** A single-fraction 14-Gy dose of local tumor irradiation followed by immunization with a single dose of 5 × 10^6^ SFVeE6,7 viral particles (*n* = 6). **d** A single 14-Gy dose of local tumor radiation combined with daily AMD3100 (3 mg/kg, i.p.) treatment (*n* = 5). **e** CXCR4-negative staining without primary antibody (negative control) and **f** H&E staining of the TC-1 tumor morphology. The brown staining represents the CXCR4 expression on tumor cells and stromal cells. All the images were acquired at × 10 magnification and × 20 in the small box. The red arrow indicates the tumor cell, and black arrow indicates the stromal cells.

In order to support our PET results, the expression of CXCR4 receptors in tumors was examined ex vivo. Immunohistochemistry showed CXCR4 receptor expression in the tumor in all groups. The intensity of staining was scored semi-quantitatively [29–31], using a 4-point scale (0, no; 1, weak; 2, moderate; and 3, strong staining). All tumors showed relatively weak to moderate staining throughout the tumor. When compared to the sham-irradiated control group, the mice treated with single-fraction radiotherapy alone showed a strong staining (15 ± 2 vs 32 ± 3, *p* < 0.01). The staining intensity further increased in the group of mice treated with the combination of single-fraction irradiation and immunization (56 ± 4, *p* < 0.001). Mice treated with single-fraction 14-Gy irradiation and AMD3100 showed moderate staining which was not statistically different from the staining intensity in sham-irradiated mice (23 ± 6 vs 15 ± 2, *p* = 0.09) (Fig. 5).

## Discussion

In the current study, we demonstrate the feasibility to monitor the effect of radiation and cancer immunization on CXCR4 receptor expression in the tumor using PET imaging. The chemokine receptor CXCR4 is overexpressed by many cancer types and associated with tumor progression and the metastatic potential of the tumor. Consequently, CXCR4 has been used as a drug target for adjuvant therapy. In addition, CXCR4 receptors are expressed by tumor infiltrating immune cells, where the CXCR4 receptors are involved in chemotaxis. Because of the roles of CXCR4 in tumor progression and immune cell trafficking, CXCR4 density in the tumor may be affected by treatment, either as a result of altered CXCR4 expression by the tumor cells, or by treatment-induced infiltration of immune cells.

In this study, we used TC-1 tumor-bearing mice to investigate the effect of treatment on CXCR4 expression. TC-1 tumors expressing the HPV E6 and E7 tumor antigens represent HPV-infected tumors, such as cervical cancer. The most common treatment for locally advanced cervical cancer involves radiotherapy and/or chemotherapy. Despite successful results obtained with these conventional treatments, many patients still show intrinsic or acquired resistance to these therapies [15–17]. In order to develop better alternatives for such patients, tumor vaccination is now under investigation as a new therapeutic approach [32–35]. In this study, we have investigated the feasibility of N-[^11^C]methyl-AMD3465 PET imaging of CXCR4 receptors density in tumor response to two way treatment strategies: conventional radiotherapy alone and radiotherapy in combination with experimental vaccination by immunization with SFVeE6,7 viral replicon particles.

We hypothesized that CXCR4-mediated chemotaxis could be involved in the immunological response to these treatments, which would likely be accompanied by an increase in CXCR4 density within the tumor. Here, we have shown that local tumor irradiation indeed caused a significant increase in the accumulation of the CXCR4 selective probe N-[^11^C]methyl-AMD3465 in the tumor, indicating that the CXCR4 density in the tumor is increased. The tracer uptake was even further increased when local single-fraction tumor irradiation was combined with immunization with a single dose of SFVeE6,7. This increase in CXCR4 density in the tumor could be due to treatment-induced increase in the secretion of CXCL12 by stromal cells. This may induce overexpression of CXCR4 by tumor cells that attempt to avoid apoptosis. Alternatively, radiotherapy-induced hypoxia induces the production of hypoxia-inducible factor-1α, which in turn stimulates the secretion of CXCL12. The enhanced CXCL12 secretion by the hypoxic tumor may lead to the recruitment of CXCR4 expressing bone marrow derived immune cells in order to restore the vasculature of the tumor after radiotherapy [14, 17, 36]. Furthermore, tumor irradiation might have resulted in increased recruitment of CXCR4-positive T lymphocytes at the tumor site as a result of the acute immune response to treatment-induced cell damage [37, 38]. In previous studies, we have shown ex vivo that local tumor irradiation indeed resulted in a strong increase in infiltrating T lymphocytes, which was further increased when radiotherapy was combined with immunization [33]. This increased influx of T-cells was accompanied by an up-regulation of chemokines and their receptors [33], which is in agreement with the result of the present study. In a previous study, we also monitored the effect of single-fraction 14-Gy local tumor irradiation in combination with SFVeE6,7 immunization and irradiation alone on the tumor infiltration of immune cells using [^18^F]FB-IL2 PET [39]. A synergistic effect of treatment on infiltrating tumor T-cells was seen, resulting in a 10-fold and 30-fold increase in tracer uptake in the tumor treated with single-fraction local irradiation alone or in combination with immunization, respectively. This may be the result of CXCR4 expressing mediated T-cell influx or CXCR4-mediated chemotaxis.

In this study, we also included a group of mice that received single-fraction tumor irradiation, followed by daily administration of the CXCR4-selective antagonist AMD3100. As a result, treatment with the CXCR4 antagonist caused a strong reduction in the tumor uptake of N-[^11^C]methyl-AMD3465 in irradiated mice. Administration of AMD3100 likely has (at least partly) saturated the binding sites of the CXCR4 receptors on tumor cells, stromal cells, and immune cells, thus preventing specific binding of N-[^11^C]methyl-AMD3465 to the receptor. In addition, inhibition of CXCR4-mediated chemotaxis by the antagonist AMD3100 could have resulted in a reduction in the radiation-induced infiltration of CXCR4-expressing immune cells into the tumor. In fact, our previous study, [^18^F]FB-IL2 PET showed that the increase in T-cells induced by radiotherapy/immunization could be partially prevented by treatment with the CXCR4 receptor antagonist [39]. Thus, the reduction in N-[^11^C]methyl-AMD3465 uptake in the tumor after treatment with AMD3100 observed in this study is likely the result of a combination of saturation of the CXCR4 receptors by the antagonist and a reduction in T-cell infiltration by inhibition of CXCR4-mediated chemotaxis.

All these data suggest that N-[^11^C]methyl-AMD3465 could detect the changes in the expression of CXCR4 receptors after radiotherapy or combination of radiotherapy/immunization. However, N-[^11^C]methyl-AMD3465 PET is not suitable for detecting the expression CXCR4 in or near organs involved in metabolism and excretion, like liver, bladder, or kidneys, because of the high background uptake of the tracer in these organs. Other tracer for CXCR4, such as [^68^Ga] pentixafor, may provide better contrast images, since this tracer is internalized, resulting in Ga-68 remaining trapped inside the cell. Thus, this tracer would reflect CXCR4 receptor turn-over and may be less suitable for assessing CXCR4 expression. The selection of the tracer, however, will usually mainly depend on availability of radioisotopes rather than the desired imaging properties. Still, our results warrant further translation of this imaging method to the studies in patients. An interesting application could be to use repetitive PET imaging with N-[^11^C]methyl-AMD3465 to investigate why some solid tumors become resistant after radio- or chemotherapy. This would provide information on the dynamic changes in receptors expression and thus the role of CXCR4 mediated chemotaxis in the development of therapy-induced resistance. Furthermore, the application of a combination of two different tracers, for example N-[^11^C]methyl-AMD3465 and [^18^F]FB-IL2, could be of interest, as it would allow simultaneous investigation of different aspects of the immune response to treatment, such as chemotaxis and T-cell activation. This information could give a more comprehensive insight in the activation and infiltration of immune cells in the tumor microenvironment.

## Conclusion

Taken together, the results from this study have demonstrated that monitoring of treatment-induced changes in CXCR4 receptor density in the tumor by N-[^11^C]methyl-AMD3465 PET is feasible. PET imaging showed that both single-fraction radiotherapy and immunization can increase the N-[^11^C]methyl-AMD3465 tracer uptake in the TC-1 tumor model. This increases in CXCR4 density is due to either increased expression of the receptors on tumor cells or increased tumor infiltration of CXCR4 expressing immune cells.
